# A Study on Data Selection for Object Detection in Various Lighting Conditions for Autonomous Vehicles

**DOI:** 10.3390/jimaging10070153

**Published:** 2024-06-22

**Authors:** Hao Lin, Ashkan Parsi, Darragh Mullins, Jonathan Horgan, Enda Ward, Ciaran Eising, Patrick Denny, Brian Deegan, Martin Glavin, Edward Jones

**Affiliations:** 1 School of Engineering, University of Galway, University Road, H91 TK33 Galway, Ireland; 2 Ryan Institute, University of Galway, University Road, H91 TK33 Galway, Ireland; 3 Valeo Vision Systems, Tuam, Co., H54 Y276 Galway, Ireland; 4 Department of Electronic and Computer Engineering, University of Limerick, Castletroy, V94 T9PX Limerick, Ireland; 5 Computer Science and Information Systems (CSIS), Faculty of Science and Engineering, University of Limerick, Castletroy, V94 T9PX Limerick, Ireland

**Keywords:** object detection, low-light conditions, computer vision, ADAS, autonomous vehicles

## Abstract

In recent years, significant advances have been made in the development of Advanced Driver Assistance Systems (ADAS) and other technology for autonomous vehicles. Automated object detection is a crucial component of autonomous driving; however, there are still known issues that affect its performance. For automotive applications, object detection algorithms are required to perform at a high standard in all lighting conditions; however, a major problem for object detection is poor performance in low-light conditions due to objects being less visible. This study considers the impact of training data composition on object detection performance in low-light conditions. In particular, this study evaluates the effect of different combinations of images of outdoor scenes, from different times of day, on the performance of deep neural networks, and considers the different challenges encountered during the training of a neural network. Through experiments with a widely used public database, as well as a number of commonly used object detection architectures, we show that more robust performance can be obtained with an appropriate balance of classes and illumination levels in the training data. The results also highlight the potential of adding images obtained in dusk and dawn conditions for improving object detection performance in day and night.

## 1. Introduction

The lighting conditions experienced by drivers change throughout the day, with variations depending on latitude. Driving under low-light conditions decreases the visibility of the environment and hence increases the probability of an accident occurring. Over 1.19 million people die each year due to road accidents, [1,2], with more than 75% of pedestrian fatalities occurring during the dark, 21% during the day, and 4% during dusk and dawn [3]. Nearly 49% of fatal traffic collisions occur during night-time, but only 25% of travel occurs during that time [4]. As autonomous driving technology advances beyond Advanced Driver Assistance Systems (ADAS), the need for highly robust and accurate object detection algorithms is continuously increasing. To achieve fully autonomous driving, the vehicle must be fully aware of its surroundings, including in different lighting conditions. In a car, the normal low beam illumination has an effective range of approximately 50 m [5,6], while the high beam illumination range is approximately 150 m. However, the stopping distance of cars traveling at high speed can be greater than 100 m in certain conditions, and therefore, there is a risk associated with detecting objects that are not sufficiently illuminated. Furthermore, this does not take into consideration the potential poor reflectivity of other vehicles and vulnerable road users (VRU), i.e., those most at risk in traffic, e.g., pedestrians and cyclists [7]. Furthermore, if the target is wearing black clothing, the pedestrian may not be seen until the distance between the vehicle and VRU is only 10 m [8]. This means that the vehicle has a very short distance and time frame to react to a potential collision, and hence there is an increased chance of accidents.

A significant challenge in night-time object detection is poor lighting, resulting in low brightness, low contrast, and noise in images. Neumann et al. [9] compared state-of-the-art pedestrian detection algorithms and found that none perform well at night, even those trained on night-time data. The failure of object detection and pedestrian detection algorithms in autonomous vehicles highlights the critical need for research in night-time object detection.

Xiao et al. [10] suggest that training models with data under normal illumination can improve performance in low-light scenarios. They found that features extracted by models trained on normal illumination differ significantly from those trained on low-light images. Their model achieved the best performance when initially pre-trained with normal illumination data, and fine-tuned with low illumination data. This suggests a complex relationship between training data composition and model performance, specifically regarding the balance between day and night-time data.

In this paper, we evaluate the impact of daytime and night-time training data on object detection performance under various lighting conditions. The aim is not to find the absolute best performance for daytime or night-time object detection but to examine the influence of image illumination in training data. Given the high costs associated with data collection, understanding the specific data requirements for training night-time object detection models is crucial. This research provides guidance on the optimal proportions of day and night data for data collection, aiming to minimize the amount of data needed and hence potentially providing some guidance that may assist in the development of improved night-time object detection systems. There is relatively little previous work on the effects of combining data from different times of the day during training. A primary goal of this paper is to address the issue of optimal data mixtures for training, particularly determining the best ratio of illumination conditions (day/night/dusk, etc.) when training data are limited.

The main contributions of this paper are (1) the analysis of the effects of using different ratios of day-to-night data in the training dataset on object detection performance in various lighting conditions, and (2) the analysis of the effects of adding dusk and dawn training data to models trained on day and night data. Experimental work uses a number of well-established neural network architectures for object detection.

The remainder of this paper starts with a presentation of related work (Section 2), where we discuss some low-light datasets that are publicly available, the technologies used to tackle low-light conditions, the object detection architectures, and the imbalance issues related to object detection. The experimental methodology used in this paper is outlined in Section 3. Section 4 analyses the results and suggests guidance as to what data combinations (day/night) could be used to create a good object detector for a given lighting condition. Finally, conclusions are drawn (Section 5), and an outlook on future work is made (Section 6).

## 2. Related Work

### 2.1. Low-Light Conditions

#### 2.1.1. Hardware

Some researchers tackle the task of object detection by implementing a two-system approach [11,12], whereby one of the systems is responsible for daytime object detection and the other is responsible for night-time object detection, to optimize performance in each condition albeit at the cost of additional complexity. Other researchers use a multi-modal approach [13,14,15,16,17,18], i.e., using other sensors to compensate for when there is a lack of information from the RGB cameras. There is a wide sensor suite available that is not reliant on visible light or the illumination of the surroundings and thus these sensors are often used in conjunction with RGB cameras. Some of the most common are Light Detection and Ranging (LiDAR) sensors, infrared sensors, radar sensors, and event-based cameras.

LiDAR works by counting the time between events in backscattered energy from a pulsed beam [19]. Because of this, LiDAR sensors are not reliant on the environment to be well illuminated, and hence, their performance in night-time scenarios is comparable to daytime performance. Many studies have been conducted to apply LiDAR in autonomous driving and night-time object detection scenarios [19,20,21], but LiDAR has the drawback of the high cost of implementation and operation. Another disadvantage of LiDAR is that it is potentially ineffective during adverse weather conditions such as heavy rain or heavy fog, as these conditions will interfere with the beams emitted by the sensor [19].

Infrared night vision systems can be divided into near-infrared (NIR), which is an active night vision system, and far-infrared (FIR)/thermal, which is a passive night vision system [6]. In NIR systems, the scene is illuminated by an NIR lamp, typically of wavelengths of 800 to 900 nm. This wavelength is not visible to the human eye and thus no dazzling effects from this light source occur. A FIR/thermal night vision system, on the other hand, does not require a light source; it passively senses electromagnetic radiation of wavelengths 8 to 14 µm, which allows the camera to see the heat signature of the scene.

These technologies are not new to the market; the first FIR thermal night vision system was released for vehicles in 2000 [22]. However, with the advancement of autonomous vehicles and the realization of the limitations of the current visual spectrum cameras, the topic of exploiting the full potential of thermal cameras has regained interest in the community, especially combining different sensors [13,16,17].

Both the NIR and FIR have their advantages and disadvantages [6]. NIR has the benefit of having a better image quality as it better resembles what is seen by a driver, but it is prone to dazzling by other light sources and requires an illuminator. FIR has the benefit of not needing an illumination source as it detects heat and can detect living objects; however, this also leads to other issues such as (1) lower resolution, and (2) objects that have the same temperature as the environment can end up being invisible. Furthermore, FIR cameras, although cheaper than LiDAR, are still more expensive than RGB cameras [23,24].

Radar is a sensor that is largely unaffected by environmental conditions. It uses the Doppler effect to measure the speed and position of targets directly. Although millimeter wave radar has good resolution, it has a short range. Micrometer wave radar, in contrast, has a longer range of 200 m but it has poor resolution [25]. Radar also has poor capability for target classification as it cannot distinguish color [26].

Event-based cameras are asynchronous sensors that sample light based on the scene dynamics, rather than synchronized by a clock. Event-based cameras have a high temporal resolution, high dynamic range, and low latency but, like radar, they do not offer detailed information such as color. They also have issues capturing static targets, as the operating mechanism is based on events/changing pixel values [27].

#### 2.1.2. Software

Traditionally, image enhancement is used to compensate for dark images. A very common approach is to enhance the contrast of the low-light images using histogram equalization [28]. Although this technique has the benefit of low computational cost, its robustness is in question as the details and information hidden in the gray levels are often lost.

Image enhancement methods based on the Retinex model manipulate the estimated illumination and then project it back to the corresponding reflectance [29,30]. However, this approach has its drawbacks as it does not include noise handling in the model. With the image enhanced, noise in the image is also enhanced, potentially leading to a lower signal-to-noise ratio. Efforts have been made to improve the Retinex model by adapting for the noise element in the images [29]. Furthermore, Xiao et al. [10] have compared the different types of image enhancement techniques and shown that although most image enhancement algorithms achieve a visually pleasing result, the resulting image does not improve the performance of object detection models. Additionally, Guo et al. [31] have achieved positive results in the area of image post-enhancement, being able to reveal many details hidden from the human eye, but again, this does not benefit machine vision algorithm performance. Kim et al. [32] trained and tested neural networks on different databases of night-time images and tested different pre-processing steps to evaluate the effect on night-time images from visible light cameras. Their results show that having variety in the training data improves performance. Their tests on the effect of histogram equalization (HE) on the model performance show minor differences of 1% accuracy between using and not using HE.

### 2.2. Object Detection

Commonly used object detection algorithms from the state-of-the-art (SotA) can be broken into two main types—one-stage detectors and two-stage detectors. Two-stage approaches, such as the Region-based Convolutional Neural Network(R-CNN) [33], mask-R-CNN [34] and Faster-R-CNN [9], have better localization and better accuracy, whereas one-stage approaches, such as YOLO [35,36,37,38] and SSD [39], achieve faster inference speed. In two-stage detectors, the first stage generates a region of interest, and the second stage classifies that region of interest. One-stage detectors propose prediction boxes directly without the region proposal step and hence are faster and more suited for real-time applications [40].

Jiao et al. [41] evaluated the performance of many SotA detectors on the COCO dataset. Their study shows that on an NVIDIA Titan X GPU, YOLO can process data at 45 Frames Per Second (FPS), whereas Fast R-CNN can only achieve 0.5 FPS.

As an example of the SotA, YOLO has evolved substantially since the original version [42]. It has experienced multiple major improvements, from accuracy improvement in YOLOv2 [9] and speed improvement in YOLOv3 [36]. In 2020, YOLOv4 [35] and YOLOv5 [38] were released. YOLOv4 and YOLOv5 have very similar performance in terms of accuracy as they share many of the same technologies in their design. For the study presented in this paper, YOLOv5 was chosen because of its relative maturity, ease of use of the PyTorch framework for model development, and short training time. For example, in one experiment carried out by Nelson et al. [43], it took 15 min for YOLOv5 to train for 200 epochs, whereas it took YOLOv4 210 min on Google Colab using the NVIDIA Tesla P100. These two versions of YOLO share similar single image inference speed (20 ms for YOLOv5 and 22 ms for YOLOv4), but due to YOLOv5’s PyTorch framework, it is easier to implement large batch inference, which is faster, i.e., 7 ms for a batch size of 36.

The development of neural network architectures continues to evolve, in particular, with the development of more efficient implementations of existing models. Wang et al. developed YOLOv7 [44] as an improved version of YOLO with a small increase in performance while having a significant decrease in size, decreasing from 64.4 M parameters to 36.9 M. Similarly, YOLOv8 [45], YOLOv9 [46] and YOLOv10 [47] are evolutions of earlier versions of YOLO. Although the small versions of these models have similar performance in terms of accuracy, significant improvements have been made regarding latency and the number of parameters. YOLOv5 and YOLOv7 have been implemented in the study in this paper due to their relative maturity and stability and comparable performance with other state-of-the-art models.

In recent years, vision transformers have gained attention and are replacing many traditional CNN-based algorithms as the new state-of-the-art for object detection tasks [48,49,50,51]. Lv et al. proposed a real-time end-to-end transformer-based detector, RT-DETR (Real-Time Detection Transformer) [52], which is used in this paper.

Table 1 shows a comparison between some of the state-of-the-art architectures from one-stage, two-stage, and transformer-based object detectors. This table shows the trade-off between architecture size and inference speed (in terms of FPS) as well as example performance results from the literature. Typically, larger architecture sizes yield better accuracy but at the cost of slower inference speed, with the exception of RT-DETR where a relatively high inference speed was achieved despite the network size.

Some researchers tackle the night-time object detection problem by refining the model architecture such that the model is more adapted toward low-light scenes. For example, Xiao et al. [10] proposed a Night Vision Detector (NVD), based on the RFB-Net, that is tailored for low illumination environments, while Wu et al. [53] proposed a method of improving object detection performance through sample selection and label extension. They also combined the architectures of multiple models to make the overall architecture more efficient. Although adapting the model architecture will improve the performance at night, this may have impacts on the model’s robustness and performance in other conditions.

### 2.3. Datasets and Data Imbalance

An objective of this paper is to examine the optimal ratio of training data from different scenarios. This sub-section examines commonly used datasets in automotive computer vision and considers the issues associated with data selection and imbalance.

Publicly available datasets such as Microsoft Common Objects in Context dataset (MS COCO) [54] are commonly used by many researchers to benchmark object detection models because these datasets offer a huge variety of classes in everyday scenes [34,35,36,55,56,57,58]. Although these datasets are widely used, many lack sufficient low-light images. For example, MS COCO has over 330 k images but less than 2% of these are low-light images [59]. Datasets such as KITTI [60], WoodScape [61], CityScape [62] and Caltech Ped [63], while widely used for automotive computer vision research, do not include annotated low-light data, which are necessary for this research.

Exclusively Dark (ExDark) is a dataset created by Loh et al. [59] that is comprised of low-light images exclusively. Although this dataset is smaller than COCO, with around 7000 images, it does offer potential for the researchers that are working on object detection under low-light conditions [64,65]. Chen et al. [66] developed the See in the Dark (SID) dataset, which provides simulated low-light images created by adjusting the camera’s exposure time and camera ISO, a measure of camera light sensitivity. While this dataset provides low-light images, they are not as natural as what an autonomous vehicle would see and the scenes in the dataset do not accurately resemble those on the road. Richter et al. [67] created a dataset of videos and images from a realistic virtual world. This dataset covers many scenarios and adverse conditions where real-world data are lacking, such as night-time, rain, and snow.

The Berkeley Deep-Drive 100K (BDD) dataset was chosen for the research in this paper. It contains almost 80,000 annotated images in total, over a range of conditions [68]. A huge advantage of this dataset is that the images vary in terms of scene, weather, classes, and time of day, and more importantly, this information is also recorded in their annotation. The time annotation in the dataset is broken into three categories: daytime, night-time, and dusk/dawn (as a single category).

In the BDD dataset, the dusk and dawn images are labeled as one category [68]. During dusk and dawn, the level of illumination is similar, with the difference of dusk going from bright to dark and dawn going from dark to bright. The definitions of day, night, dusk, and dawn are often very subjective. The scientifically accepted definition of dusk and dawn is the period where the center of the sun is between 0 and 18 degrees below the horizon. While the sun is above the horizon it is day and when it is 18 degrees below the horizon it is night [69].

The images from the BDD dataset are all dashcam images, similar to data that would be obtained and used in autonomous vehicles. This makes it even more suitable for the training of autonomous vehicle applications. The dataset is comprised of over 100 k videos obtained from vehicle-mounted sensors. The BDD dataset has been used by many researchers [53,70,71,72] to develop object detection algorithms and image enhancement for night-time and low-light applications, as it is one of the biggest open source datasets that cover diversity in classes, scenes and time of day. A further reason why BDD was chosen for this study is that it contains night-time and dusk/dawn data, whereas many of the datasets mentioned above do not offer this data.

Icanu et al. [73] have performed experiments with different combinations of training datasets to study object detection in night-time traffic scenes. Multiple datasets were used in [73], including BDD [68], VIPER [67], GTSRD [74], and CVL [75], with over 220 k images in total. They compared the performance increase of a YOLOv3 deep neural network model [36] pre-trained on the MS COCO dataset with the performance of that same model after adding different datasets to the training data.

Although some previous studies have combined different day and night image datasets in an attempt to improve object detection performance [13,75], the question of what is the optimal balance between day and night images during the training of an object detector remains unanswered. Different imbalance-related issues can occur during object detection [76,77,78,79]; when left unaddressed, these issues can greatly impact the performance of object detection [76]. One particularly important imbalance is the class imbalance, which occurs when there is a significant inequality among the number of examples pertaining to different classes, where some classes are over-represented and others are under-represented [76]. This can be further broken into foreground–foreground imbalance (a small subset of classes dominates the dataset) and foreground–background imbalance (background instances outnumber the positive foreground instances). Of these two types of class imbalances, foreground–background is inevitable as there will always be more background objects than foreground objects in the datasets. The solution to address this issue is usually integrated into the functionality of the model. For example, YOLO uses a soft sampling technique called focal loss, in which the class loss values are adjusted dynamically [57]. Other solutions include hard sampling techniques such as random sampling, in which a fixed number of positive and negative samples of foreground and background are extracted as a set of anchors for each image.

Foreground–foreground imbalance refers to situations where there are classes in the foreground that significantly outnumber another class in the foreground. An example of this may be substantially more cars than trailers occurring in an urban dataset. The solution to this problem often involves manual intervention rather than being addressed by the model design. Approaches include fine-tuning the model [80], image weighting during training [81,82], and Online Foreground Balancing (OFB) [83].

## 3. Methodology

**Figure 1:**
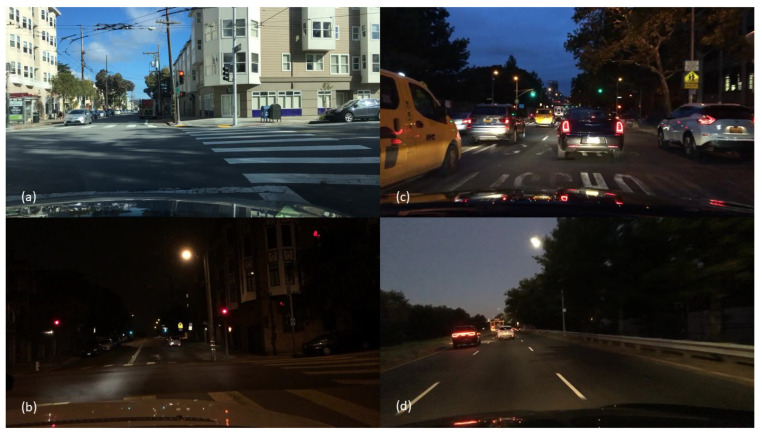
Example footage from the BDD dataset. (**a**) Day, (**b**) Night, (**c**) Dawn, (**d**) Dusk.

This section outlines the methodology used for the research described in this paper. As shown in Table 2, the BDD dataset contains almost 80,000 images, of which approximately 42,000 are day images, 32,000 are night images and 6000 are dusk and dawn images. Figure 1 shows some image examples from the BDD dataset. In order to isolate the effects of changing the image ratios, we take into consideration the class balancing, class count, and image count during the preprocessing stage.

### 3.1. Metrics

The key metric used during the evaluation of the performance of the models is the Mean Average Precision (mAP) [84]. Other metrics discussed here include Recall, Precision, True Positive Rate, and True Negative Rate. A True Positive (*TP*) is the correct detection of a ground-truth bounding box. A False Positive (*FP*) is an incorrect detection of a nonexistent object or a misplaced detection of an existing object. A False Negative (*FN*) is an undetected ground-truth bounding box [85].

Precision is the ability of a model to identify only relevant objects. As shown in Equation (1), it is the percentage of true positive predictions, amongst all predictions. Recall is the ability of a model to find all relevant cases (all ground-truth bounding boxes). It is also called the True Positive Rate or Sensitivity. As shown in Equation (2), it is the ratio of true positive predictions among all given ground truths. The True Negative Rate (*TNR*) (also called Specificity) is calculated as shown in Equation (3). This is the probability that an actual negative case will be correctly classified.

(1) $$Precision=\frac{TP}{\left(TP+FP\right)}=\frac{TP}{\left(AllDetections\right)}$$

(2) $$Recall=\frac{TP}{\left(TP+FN\right)}=\frac{TP}{\left(AllGroundTruth\right)}$$

(3) $$TNR=\frac{TN}{\left(TN+FP\right)}$$

The mAP is obtained by averaging the area of the Precision/Recall curve for each category [86]. The Precision/Recall curve captures the trade-off between precision and recall. A high area under the curve represents both high precision and recall, where high precision corresponds to a low false positive rate and high recall corresponds to a low false negative rate. The mAP(50) used in this study is based on an intersection over union (IOU) value of 0.5, such that the predicted object bounding box overlaps by 50% or more with the ground truth bounding box. Similarly, mAP(50:95) is the average mAP over different IOU thresholds, from 0.5 to 0.95, in steps of 0.05. By increasing the range of IOU thresholds, mAP becomes a more challenging metric. MS COCO [54] further considers mAP according to object size since object size affects performance directly, as smaller objects are generally harder to detect. Objects are divided into three categories: Small, Medium, and Large. Small objects are objects of size 32 × 32 pixels or smaller. Medium objects are between the size of 32 × 32 and 96 × 96 pixels. Large objects are objects above 96 × 96 pixels. These object size categories give rise to the following metrics: AP(small), AP(medium), and AP(large) [54]. More specifically, mAP(50:95)(small), mAP(50:95)(medium) and mAP(50:95)(large) are presented in this paper. Again, averaging mAP over a range of IOU thresholds for different sizes of objects provides a more challenging performance measure.

### 3.2. Pre-Processing

The BDD dataset has an uneven class distribution, which will lead to bias and poor performance of the model if not accounted for. As shown in Table 3, the number of instances of each class used in training is reflected in the validation and evaluation, thus maintaining approximately the same class ratio. This is performed to minimize any change in performance caused by class imbalance and underrepresented data between the training, validation, and evaluation datasets.

Even with the efforts made to address class imbalance, there can still be significant variation in the rate of occurrence of classes in practice; for example, classes such as trains and trailers appear naturally at a lower rate in the real world. This presents challenges in terms of having sufficient examples for training (separate from the class imbalance problem). In this study, classes with less than 1000 instances are removed and are not considered during the training of the model; these include trains and trailers with only 143 and 73 instances, respectively, in the 80,000 images in the dataset.

Another approach used to address the class imbalance issue is via the image weighting function within YOLOv5 [38]. This function samples images from the training set weighted by their inverse mAP from the previous epoch’s testing, rather than sampling the images uniformly as in normal training. This results in images with a high content of low-mAP objects being selected with a higher likelihood during training.

### 3.3. Training and Evaluation

Initial performance evaluation was carried out using the YOLOv5 small architecture [38]. As noted above, this architecture was chosen because it is a mature, relatively small model, with a fast inference time; this makes it a closer approximation to a model implemented in an automotive application. Using the extra-large YOLO model gives less than 10% mAP improvement but the training time and the number of parameters increase by more than a factor of 10. As shown in Table 4, models were trained using 5 different image subsets of different day/night ratios, while maintaining the same CNN architecture. The image sets used for training are differentiated by the ratio of day-to-night images each contains, e.g., D100N00 contains 100% daytime images and 0% night-time images. Each image set contains 32,000 images in total. Each model was trained from scratch, for 50 epochs with a learning rate of 0.01, using Nvidia Tesla T4 GPUs with a batch size of 32. Other hyperparameters are set to the framework’s default configurations [87].

**Figure 2:**
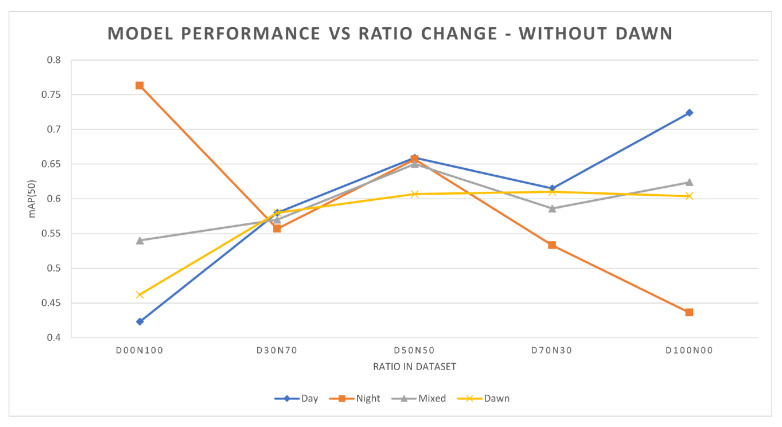
Performance of YOLOv5s models trained with different day-to-night image ratios, without dusk/dawn in the training data. Four evaluation subsets from the different times of day it was used.

Table 5 shows the mAP(50) performance for YOLOv5 for different ratios of training data, without dusk/dawn images in the training data. Figure 2 plots the mAP(50) values as a function of the percentage of daytime data in the training set. The training and validation subset ratios are as before. Four different evaluation subsets were used, each comprised of 5000 images. The four subsets were day, night, a mixture of day and night (mixed), and dusk/dawn. Each trained model was evaluated using the 4 evaluation datasets. Bootstrap sampling was performed during the evaluation of the models, in which the standard error for all the models trained was less than 0.005.

The training and evaluation process described above that was performed on the YOLOv5s architecture was repeated with Faster-RCNN [9], YOLOv7n [44] and RT-DETR (Real-Time Detection Transformer) [52] architectures, as further representative architectures from the 2-stage, 1-stage, and transformer-based object detection algorithms. Results for these other model architectures are discussed below.

**Figure 3:**
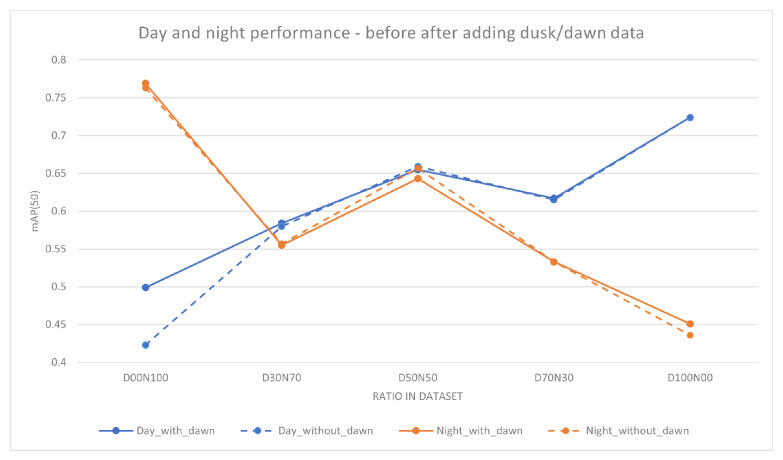
Performance comparison of YOLOv5s models before and after the addition of dusk/dawn data. The X-axis is the ratio of the day-to-night images used in the training subset and the Y-axis is the mAP(50) of the models. The dashed lines are the models trained without dusk/dawn data and the solid lines are models trained with added dusk/dawn data. Adding dusk/dawn has positive effects on the performance of the models, with the largest difference at D00N100.

A further investigation was conducted by adding dusk and dawn data into the training. This is performed to investigate the effects of dusk and dawn data on the model performance for each day-to-night ratio. A further approximately 3500 images were added to each image set. The amount of dusk/dawn images added is not the same in every model because the class balance within the subset was maintained. A change in the class count can lead to a change in performance unrelated to the illuminance of the image. YOLOv5s was trained with added dusk/dawn data, and the resulting model was evaluated using the four evaluation subsets mentioned above, i.e., day, night, mixed, and dusk/dawn. Performance using mAP(50) for this experiment is shown in Table 6. Figure 3 graphically illustrates the differences in performance (mAP(50)) as a result of adding dusk/dawn data.

Dusk and dawn images are of particular interest because the illuminance level falls between the well-illuminated daytime images and the poorly illuminated night images. When discussing day images, it is generally assumed to mean well-illuminated images but not all daytime images are well-illuminated, as there may be other factors during the day that may impact image quality. An example of such a factor would be shadows; a shadow that is cast on an object can make the object seem very dark even though it is daytime. Likewise, at night-time, there are factors that can improve the image quality, like artificial light sources, including car lights and street lights. However, the illumination at night-time is, in general, poorer than during the daytime.

## 4. Results

### 4.1. Experimental Results

**Figure 4:**
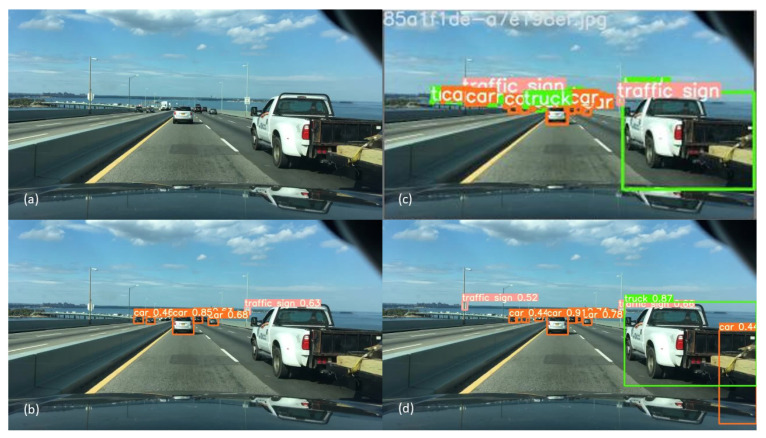
Example showing the effect of training with dusk/dawn data. (**a**)—Original image, (**b**)—D00N100 without dusk/dawn, (**c**)—ground truth, (**d**)—D00N100 with dusk/dawn (correctly identifies truck).

On the basis of the results in Table 5 and Table 6, as well as Figure 2 and Figure 3, a number of points emerge:

1. Firstly, as expected, it can be seen that when increasing the percentage of daytime training data, the daytime object detection performance will generally increase. Similarly, the same result can be seen for night-time data (Table 5). The total number of images used in each model is the same, and the number of instances of each class is kept as close as possible between the models. This suggests that the increased percentage of daytime training images is giving the model new and useful information, which is reflected in the increase in performance.
2. Secondly, when the model is initially trained with only day or night data, adding a small amount of the missing data will give a significant increase in performance for the category of the missing data. When the model is trained with solely one category of image (day or night) the performance in that category is better than that of other mixed training. As shown in Table 5, D00N100 and D100N00 perform best in the night and day categories, respectively. However, when there is a small amount of training data of the other category (day or night) added then there is a significant increase in the mAP for that category. As shown in Table 5 for the model D00N100, by replacing 10,000 images from night to day, the performance for day increased by 16%. Similarly for the D100N00 model, the replacement of 10,000 images from day to night improved the night-time performance by 10%.
3. Thirdly, as shown in Figure 2 and in Table 5, the increase in the performance is not linear, which means simply increasing the amount of data may not yield the best results. For example, D50N50 outperforms D70N30 during the day even though D50N50 has less daytime training data. A similar situation can be seen when comparing D50N50 and D30N70 during night-time performance. This suggests that models may benefit more from carefully selected training data.
4. Fourthly, the addition of dusk and dawn data will improve the day and night performance. As shown by examining Table 5 and Table 6, there was an improvement across the board with the addition of a small amount of dusk and dawn data. The highest increase was found in the D00N100 model, with an increase of 7.6% in mAP. The impact of this can be seen through the example in Figure 4, where the model that was trained with dusk and dawn data was able to detect the truck in the image, while the model without dusk and dawn was not able to detect this. For the objects that both models can detect, the model trained with dusk and dawn data performs the detection with higher confidence. The largest boost in performance from adding dusk/dawn data occurs when the training subset is initially comprised of only day or night, as shown in Figure 3. Although there was less of an increase in the other models, it still shows that dusk/dawn acts as a useful bridge between day and night images.
5. Lastly, the best overall performance where robustness across different scenarios is the goal is achieved when there is a balanced mixture of data. Table 7 shows mAP(50) for a range of model architectures other than YOLOv5, and it can be seen that the same trend is seen across the different architectures. Although a model may have better performance specifically at day or at night if trained with only day or night data, respectively, there is a loss of robustness in contrary conditions. The optimal ratio used in the training data will depend on the specific end goal and use case of the model.

The behaviour seen in the mAP(50) results for YOLOv5s in Table 5 and Table 6 are largely replicated in Table 8 and Table 9, which show more detailed results using mAP(50:95), mAP(50:95)(small), mAP(50:95)(medium), and mAP(50:95)(large), with all metrics showing essentially the same trends.

Using the correct balance in the training dataset means the training process becomes more efficient in terms of the quantity of training data required, which in turn requires less training time. The performance of the models generated in this study is comparable to other similar studies often with larger datasets. Unger et al [75] used a combination of four datasets, with a total of 137,000 images for training, with a resulting mAP of 0.63. Iancu et al [73] used 65,000 images from the BDD dataset to achieve a performance of 0.63, on YOLOv3. The results presented in this study achieved similar performance with only 32,000 images used for training.

### 4.2. Considerations for Data Selection

The main objective of this paper was to examine the impact of data selection on computer vision performance in low-light conditions, and in particular, the optimal ratio of training data captured in different conditions (day, night, dusk). While absolute performance depends on the specific model and dataset used, there are some broad points of guidance that emerge:

- **Ensure Data Distribution Matches the Use Case**: While not specific to the low-light condition, the class imbalance problem is important (and is well-known). The class distribution of the training data should align with the use case in the training, validation, and evaluation subsets.
- **Use Training Data from the correct domain**: For models intended for daytime use, only daytime training data should be employed. For models intended for night-time use, employ only night-time training data. For example, in this study, as shown in Section 4.1, the D100N0 training subset performs the best during the day and D00N100 performs the best at night.
- **Balance Training Data Across Multiple Domains**: If the model is designed for use across multiple domains, ensure that the training data are balanced across these domains. Section 4.1 shows that an even split (D50N50) performs the best when working across domains.
- **Incorporate Dusk and Dawn Data**: Especially for models intended for night-time or multi-domain use cases, data from dusk and dawn in the training dataset enhances performance across varying lighting conditions.

## 5. Conclusions

As technology develops and moves from ADAS toward fully autonomous vehicles, the drive for improvement in computer vision also grows. Object detection, being a key part of computer vision in autonomous driving, is required to be highly precise and efficient while also being robust. Research has contributed to improving the accuracy and speed of object detection through architectural and algorithmic improvements. However, the robustness of the model across multiple scenarios is often overlooked. Object detection performance will depend heavily on the training data, and the selection of training data will depend on the final application. A critical factor is the lighting conditions in the environment. While concentrated training on one lighting condition will create a model that performs well in that lighting condition, this will also make the model lose its robustness when exposed to other lighting conditions.

This study has investigated several issues regarding object detection under low-light conditions. Issues regarding class imbalance were identified, and some solutions to address these issues have been outlined. This study has shown the importance of class balancing and the increase in performance after addressing this is shown in the results. Also shown are the effects of training a model with both day- and night-time images and the impact of this on the performance of the model. The same trends are seen across the multiple model architectures considered, which suggests that the difference in performance between models is largely due to the change in the day-to-night image ratio in the training data. Naturally, the absolute performance of a model will depend on the architecture implemented and the database used, however, we believe that the trends established in this paper should generalize to other models. Finally, the study highlights the potential of dusk and dawn images for improving the performance of both the day and night performance of the model. In addition, some common problems encountered during the training of a neural network, including dataset selection, and class imbalance are discussed, thereby providing an understanding of the obstacles encountered during the pruning of a dataset and the selection of the data for training. The results lead to some guidance on what data could be used to create a good object detector for a given lighting condition.

## 6. Future Work

A large dusk/dawn dataset would allow the full potential of dusk and dawn data in improving performance to be explored. Furthermore, a large dataset with illumination information, such as the lux level for each image, in addition to the typical environment data would be greatly beneficial for research in this area. The illuminance of an image (and objects within the image) will vary due to many factors; time of day and position of the sun are just some of the more common ones, while other factors such as artificial lighting and shadows are also very impactful. A larger dataset that contains the illumination information and a greater variety in illuminance will allow a finer categorization of the data and will allow the possibility of exploring what information the images at each illumination level are providing to the training model and the resulting impact on performance.

Another approach that can be considered is the use of simulations to generate the data and scenarios required to further investigate this topic, though this is not as desirable as real-world data. However, the use of simulation to generate the required data would lower the cost of data collection of real-world data, though an adequate simulation would require an accurate simulation of the camera sensor, camera lens, noise model, and environmental light ray tracing.

## Figures and Tables

**Table 1:** Comparison of the number of parameters (# Parameters), inference speed (FPS), and average precision (AP) (tested on the COCO dataset) of the architectures used [44,49,52].

Architectures	# Parameters (M)	Frame per Second (FPS)	${\mathrm{AP}}^{\mathrm{COCO}}$
YOLOv5s	7.2	156	37.4
YOLOv7n	6.2	286	38.7
DETR	41	28	43.3
RT-DETR	32	114	53
Faster-RCNN	166	16	39

**Table 2:** Breakdown of BDD dataset.

Time of Day	Label Count
Day	41,986
Night	31,900
Dusk/Dawn	5942
Total	79,828

**Table 3:** Instances of each class used in each dataset.

Classes	Each Training Dataset	Each Validation Dataset	Each Evaluation Datasets	Classes (As a % of Total)
Car	578,549	63,699	123,206	55.006
Traffic Sign	193,419	21,840	44,097	18.688
Traffic Light	138,272	17,180	42,951	14.701
Pedestrian	76,020	8447	14,548	7.228
Truck	23,137	2571	5049	2.200
Bus	9819	1091	1902	0.934
Bicycle	5877	653	1175	0.559
Rider	3757	417	745	0.357
Motorcycle	2508	279	533	0.238

**Table 4:** Image ratio used in each model.

Ratio Name	Day Percentage	Night Percentage	Image Count
D100N00	100	00	32,000
D70N30	70	30	32,000
D50N50	50	50	32,000
D30N70	30	70	32,000
D00N100	00	100	32,000

**Table 5:** Different data mixtures used to train each model, **without** dusk/dawn, and the mAP(50) results on each of the four evaluation subsets.

	Training Data (Image Count)			Evaluation Results, without Dawn (mAP(50))			
Ratio	Day	Night	Dusk/Dawn	Day	Night	Mixed	Dusk/Dawn
D00N100	0	31,890	0	0.423	0.763	0.540	0.462
D30N70	9850	22,982	0	0.580	0.557	0.570	0.580
D50N50	16,416	16,416	0	0.659	0.657	0.650	0.607
D70N30	22,982	9850	0	0.615	0.533	0.586	0.610
D100N00	31,890	0	0	0.724	0.436	0.624	0.604

**Table 6:** Different data mixtures used to train each model, **with** dusk/dawn, and the mAP(50) results on each of the four evaluation subsets.

	Training Data (Image Count)			Evaluation Results, with Dusk/Dawn (mAP(50))			
Ratio	Day	Night	Dusk/Dawn	Day	Night	Mixed	Dusk/Dawn
D00N100	0	31,890	3520	0.499	0.769	0.586	0.647
D30N70	9850	22,982	3721	0.584	0.555	0.570	0.695
D50N50	16,416	16,416	4278	0.655	0.643	0.644	0.710
D70N30	22,982	9850	2383	0.617	0.533	0.585	0.617
D100N00	31890	0	3520	0.724	0.451	0.628	0.611

**Table 7:** Performance (mAP(50)) of different architectures trained on different ratios. Similar trends at each ratio can be seen across the different model architectures, as observed with YOLOv5S in Table 5, Table 6, Table 7, Table 8 and Table 9.

	YOLOv5s			Faster-RCNN			RT-DETR			YOLOv7n		
Ratios	Day	Night	Mixed	Day	Night	Mixed	Day	Night	Mixed	Day	Night	Mixed
D00N100	0.423	0.763	0.54	0.419	0.676	0.504	0.465	0.591	0.509	0.438	0.553	0.482
D30N70	0.58	0.557	0.57	0.477	0.454	0.469	0.541	0.522	0.531	0.55	0.535	0.54
D50N50	0.659	0.657	0.65	0.557	0.559	0.56	0.556	0.543	0.546	0.583	0.556	0.57
D70N30	0.615	0.533	0.586	0.508	0.454	0.489	0.507	0.463	0.49	0.585	0.525	0.563
D100N00	0.724	0.436	0.624	0.62	0.383	0.536	0.563	0.443	0.516	0.611	0.45	0.551

**Table 8:** Performance of YOLOv5s for mAP(50:95)_ALL_(combination of small, medium and large objects), mAP(50:95)(small), mAP(50:95)(medium), mAP(50:95)(large), trained **without** dusk/dawn images.

	mAP(50:95)_ALL_				Evaluation Result mAP(50:95)(Small)				Evaluation Result mAP(50:95)(Medium)				Evaluation Result mAP(50:95)(Large)			
Ratio	Day	Night	Mixed	Dusk/Dawn	Day	Night	Mixed	Dusk/Dawn	Day	Night	Mixed	Dusk/Dawn	Day	Night	Mixed	Dusk/Dawn
D00N100	0.211	0.447	0.287	0.227	0.088	0.209	0.117	0.105	0.274	0.496	0.342	0.288	0.362	0.595	0.474	0.385
D30N70	0.303	0.286	0.30	0.303	0.143	0.127	0.139	0.14	0.371	0.309	0.351	0.375	0.504	0.435	0.497	0.486
D50N50	0.366	0.357	0.355	0.314	0.182	0.159	0.166	0.146	0.444	0.401	0.413	0.391	0.578	0.508	0.548	0.513
D70N30	0.325	0.271	0.305	0.326	0.154	0.107	0.138	0.146	0.402	0.298	0.362	0.404	0.532	0.417	0.514	0.521
D10N00	0.411	0.218	0.352	0.317	0.199	0.08	0.162	0.149	0.493	0.246	0.42	0.388	0.653	0.356	0.546	0.533

**Table 9:** Performance of YOLOv5s for mAP(50:95)_ALL_(combination of small, medium and large objects), mAP(50:95)(small), mAP(50:95)(medium), mAP(50:95)(large), trained **with** dusk/dawn images.

	mAP(50:95)_ALL_				Evaluation Result mAP(50:95)(Small)				Evaluation Result mAP(50:95)(Medium)				Evaluation Result mAP(50:95)(Large)			
Ratio	Day	Night	Mixed	Dusk/Dawn	Day	Night	Mixed	Dusk/Dawn	Day	Night	Mixed	Dusk/Dawn	Day	Night	Mixed	Dusk/Dawn
D00N100	0.254	0.463	0.316	0.362	0.111	0.227	0.133	0.184	0.319	0.510	0.368	0.438	0.435	0.619	0.529	0.545
D30N70	0.311	0.278	0.295	0.393	0.145	0.118	0.142	0.194	0.384	0.306	0.342	0.474	0.523	0.426	0.488	0.627
D50N50	0.366	0.359	0.359	0.411	0.182	0.157	0.170	0.199	0.447	0.405	0.420	0.500	0.569	0.499	0.576	0.632
D70N30	0.330	0.271	0.306	0.326	0.159	0.119	0.145	0.148	0.405	0.298	0.36	0.408	0.53	0.427	0.486	0.534
D100N00	0.411	0.218	0.344	0.323	0.201	0.080	0.166	0.144	0.496	0.253	0.413	0.401	0.654	0.342	0.539	0.552

## Data Availability

The original contributions presented in the study are included in the article, further inquiries can be directed to the corresponding authors.
